# Biomechanical, structural and biological characterisation of a new silk fibroin scaffold for meniscal repair

**DOI:** 10.1016/j.jmbbm.2018.06.041

**Published:** 2018-10

**Authors:** Daniela Warnecke, Svenja Stein, Melanie Haffner-Luntzer, Luisa de Roy, Nick Skaer, Robert Walker, Oliver Kessler, Anita Ignatius, Lutz Dürselen

**Affiliations:** a Institute of Orthopaedic Research and Biomechanics, Centre for Trauma Research Ulm, Ulm University Medical Centre, Helmholtzstr. 14, 89081 Ulm, Germany; b Orthox Ltd., Abingdon, UK; c Centre of Orthopaedics and Sports, Zurich, Switzerland; d University Medical Centre, Clinic for Orthopaedic Surgery, Magdeburg, Germany

**Keywords:** Meniscus replacement, Silk scaffold, Biomechanical tests, µ-CT

## Abstract

Meniscal injury is typically treated surgically via partial meniscectomy, which has been shown to cause cartilage degeneration in the long-term. Consequently, research has focused on meniscal prevention and replacement. However, none of the materials or implants developed for meniscal replacement have yet achieved widespread acceptance or demonstrated conclusive chondroprotective efficacy.

A redesigned silk fibroin scaffold, which already displayed promising results regarding biocompatibility and cartilage protection in a previous study, was characterised in terms of its biomechanical, structural and biological functionality to serve as a potential material for permanent partial meniscal replacement. Therefore, different quasi-static but also dynamic compression tests were performed. However, the determined compressive stiffness (0.56 ± 0.31 MPa and 0.30 ± 0.12 MPa in relaxation and creep configuration, respectively) was higher in comparison to the native meniscal tissue, which could potentially disturb permanent integration into the host tissue. Nevertheless, µ-CT analysis met the postulated requirements for partial meniscal replacement materials in terms of the microstructural parameters, like mean pore size (215.6 ± 10.9 µm) and total porosity (80.1 ± 4.3%). Additionally, the biocompatibility was reconfirmed during cell culture experiments. The current study provides comprehensive mechanical and biological data for the characterisation of this potential replacement material. Although some further optimisation of the silk fibroin scaffold may be advantageous, the silk fibroin scaffold showed sufficient biomechanical competence to support loads already in the early postoperative phase.

## 1 Introduction

The menisci are two crescent-shaped fibrocartilaginous structures, located between the femur and tibia in the knee joint. Their crucial role in load bearing and distribution of contact load over the articular surfaces, achieved by a synergy of their geometry, unique material properties and the anterior and posterior attachments to the tibia plateau, is well established. Furthermore, the menisci are involved in joint stabilisation and lubrication (Bullough et al., 1970, Masouros et al., 2010, Brindle et al., 2001, Mow and Huiskes, 2005).

During everyday activities, high loads occur in the knee joint, with resultant forces of 2–3.5 times body weight (Kutzner et al., 2010). Thereby, up to 81% of the axial forces are transferred through the menisci (Pena et al., 2005). Being subjected to these high mechanical stresses, the menisci are particularly prone to injury. In total, 37% of all sports-related injuries are knee-joint related. Lesions of the medial meniscus are the second most frequent internal knee injury (24%), requiring surgical intervention in approximately 80% of the cases (Majewski et al., 2006). The most commonly performed procedure to treat a torn meniscus is partial meniscectomy, initially combining the advantage of rapid pain relief and restoration of joint function (Hede et al., 1992, Schimmer et al., 1998). Nevertheless, meniscectomy can cause degeneration of the articular cartilage in the long-term (Hede et al., 1992, Baratz et al., 1986, Seitz et al., 2012, Fairbank, 1948, Roos et al., 1998, Englund and Lohmander, 2004). This is due to the fact that a decreased contact area after meniscal resection leads to increased stress on the articular surface (Fukubayashi and Kurosawa, 1980), which becomes greater with increased removal of meniscal tissue (Baratz et al., 1986, Lee et al., 2006, Ahmed and Burke, 1983).

Consequently, there has been an increased awareness of meniscus preservation or replacement techniques in recent years. Between 2005 and 2011, the number of repairs performed increased by 11.4% for isolated meniscus tears (Abrams et al., 2013). However, irreparable lesions in the avascular region of the meniscus require partial or even total meniscectomy. In these cases, natural (e.g. allografts) or synthetic meniscal substitutes are options to restore meniscal function. To successfully replace an injured meniscus, Stone et al. once postulated basic requirements for meniscal replacement materials (Stone, 1996, Stone et al., 1997), which were later further elaborated by Rongen et al. (2014). The authors stated that the biomechanical properties of a substitute should mimic that of native meniscal tissue as close as possible. Thereby, transmitting and distributing loads over the articulating surfaces and reducing peak stresses also already in the initial phase after implantation (Stone, 1996, Stone et al., 1997, Rongen et al., 2014). Additionally, the friction coefficient should not exceed 0.05 to prevent early cartilage abrasion. Furthermore, a meniscal scaffold is thought to serve as a “framework”, encouraging cell adhesion and differentiation, vascularisation and matrix deposition. Therefore, macropores (200–300 µm), interconnected via micropores (10 – 50 µm) and a high total porosity (≥ 70%) are demanded (Rongen et al., 2014). This is particularly true for a partial replacement device, for which a connection to the remaining host tissue is essential. Many studies have evaluated the suitability of different artificial materials for meniscal replacement, but none have clinically demonstrated the capacity to protect the articular cartilage (Rongen et al., 2014, Rodkey et al., 1999, Buma et al., 2004, Kohn et al., 1992, Walsh et al., 1999, Bruns et al., 1998, Gastel et al., 2001, Peters and Wirth, 2003, Verdonk et al., 2005, Milachowski et al., 1990, Noyes and Barber-Westin, 2005). Nevertheless, two alloplastic scaffolds for partial meniscal replacement are clinically available (CMI^®^, Collagen Meniscus Implant, Ivy Sports Medicine, Gräfelfing, Germany and Actifit^®^, Orteq Ltd., London, UK) but have not gained widespread clinical adoption and their ability to protect the articular cartilage in the long-term remains unclear (Rongen et al., 2014). Sandmann et al. (2013) additionally had shown that the mechanical/viscoelastic properties of both replacement materials were significantly different to that of human menisci. Consequently, there is still a need for an adequate replacement material. Further approaches, but primarily for total meniscal replacement, are in preclinical development (e.g. Meniscofix™, Novopedics Inc. (Balint et al., 2012, Merriam et al., 2015, Patel et al., 2016) or NuSurface®, Active Implants Ltd. (Elsner et al., 2010; Shemesh et al., 2014; Zur et al., 2011)). Another non-resorbable scaffold based on silk fibroin (FibroFix™, Orthox Ltd., Abingdon, UK), designed for partial meniscal replacement, was previously tested by us in a sheep model and displayed superior results in comparison to partial meniscectomy after six months of implantation with compressive properties in the range of meniscal tissue and evidence of a chondroprotective effect (Gruchenberg et al., 2015). However, fixation and integration into the adjacent meniscal tissue was insufficient. Therefore, the material was subjected to an optimisation process and a silk fibre mesh was integrated into the porous matrix to improve anchoring of the fixation sutures. Recently, we investigated the frictional properties of this second generation of silk fibroin scaffolds (Warnecke et al., 2017). The scaffold, in comparison to the physiologically articulating surfaces of the meniscus and articular cartilage, displayed slightly higher friction coefficients than the native meniscus (Warnecke et al., 2017), but still remaining within the range of the mentioned requirements (Stone, 1996, Stone et al., 1997, Rongen et al., 2014).

Having these requirements in mind, the aim of the present study was to characterise the biomechanical, structural and biological properties of the second generation of silk fibroin scaffolds for partial meniscal replacement.

## 2 Material and methods

### 2.1 Study design

To characterise the biomechanical and structural properties, as well as the biocompatibility of the silk fibroin scaffold (FibroFix™ Meniscus, Orthox Ltd.), several tests were performed on specimens of standardised geometry. Silk fibroin flat sheet scaffolds were delivered by Orthox Ltd., which were materially and structurally identical to the meniscus implants (FibroFix™ Meniscus, ORTH REP M081), differing only in the final shape.

The study comprised testing procedures, including tensile, indentation, unconfined compression creep and relaxation tests, to determine the viscoelastic material parameters of the scaffold. Here, the ultimate tensile force *F*_*max*_ in N, the linear elastic modulus *E* in MPa, the residual force *F*_*res*_ in N and the equilibrium moduli *E*_*eq*_ in MPa in compression creep and the relaxation configuration were determined. Additionally, a dynamic mechanical analysis (DMA) was included to characterise the viscous (damping factor tan*(δ)* and loss modulus *E’* in MPa) and elastic properties (storage or elastic modulus *E′* in MPa) in greater detail.

The microstructure and morphology of the silk fibroin scaffold were assessed by micro-computed tomography (µ-CT). Thereby, total porosity and pore size were evaluated. Finally, biocompatibility was determined by MTT and BrdU tests for cell metabolism and proliferation, respectively.

### 2.2 Material

Silk fibroin scaffolds were manufactured from commercially obtained *Bombyx mori* silk fibroin fibres (Silk Opportunities Ltd, Volketswil, Switzerland). Raw fibres were degummed according to Gulrajani et al. (1996) and dissolved in lithium bromide before transferring the resulting solution to semi-permeable moulding vessels. These contained organised fibroin fibre layers comprising braided fibroin threads of approximately 0.4 mm gauge arranged in orthogonal meshes to improve anchoring of sutures used in vivo for fixation of the scaffold to the host tissue. To ensure preservation of these mesh elements, moulding vessels were rapidly dialysed against excess ultrapure water, before perfusion with a dilute acidic solution to initiate transition of fibroin to the β-pleated-sheet conformation. A macroporous internal structure was introduced through a freeze-thaw cycle. Afterwards, the scaffolds were dehydrated to maximise β-pleated-sheet content in the fibroin and increase fibroin crystallinity, before washing in ultrapure water and final transfer to phosphate-buffered saline (PBS).

### 2.3 Mechanical tests

#### 2.3.1 Test setup

All biomechanical tests were performed at room temperature (20–22 °C). Care was taken to keep specimens moist in PBS during sample preparation as well as during testing using custom-made testing chambers filled with PBS for indentation-, unconfined compression creep and –relaxation tests.

##### 2.3.1.1 Quasi-static testing

The quasi-static tests were performed using standard materials testing machines (Z 010 or BXE-EZ001.A50-000, Zwick & Roell, Zwick GmbH & Co. KG, Ulm, Germany) associated with the Zwick^®^ test software TextXpertII for testing and data acquisition.

**Fig. 1:**
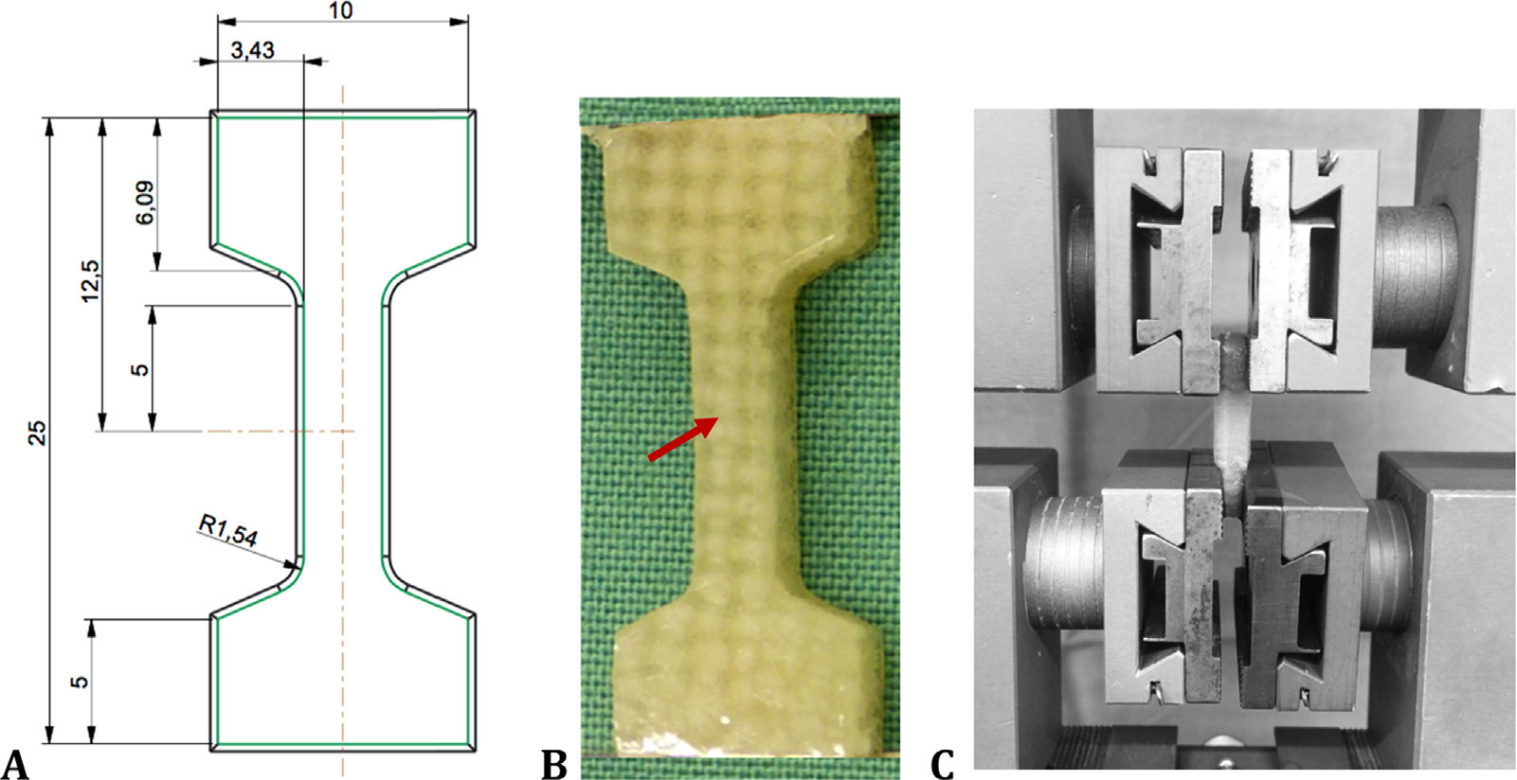
Dimensions of a custom-made punch (A) to obtain dumbbell-shaped samples from a flat sheet of the silk fibroin scaffold (B). Here, special care was taken that at least one longitudinal fibre bundle was within the tapering of the sample (red arrow, B). Afterwards, it was clamped within a standard materials testing machine (Z 010, Zwick & Roell, Ulm, Germany) using standard clamps for testing the tensile properties (C). (For interpretation of the references to color in this figure legend, the reader is referred to the web version of this article.)

**Tensile test to failure:** The tensile properties of the silk fibroin scaffold were determined in a tensile test to failure, performed according to Villegas et al. (2007). Dumbbell-shaped specimens (n = 9, approximately 25 mm × 10 mm × 5 mm) were cut from each silk fibroin flat sheet using a custom-made punch and mounted in the materials testing machine equipped with a 500-N load cell (KAF-W, A.S.T GmbH, Blaustein, Germany, accuracy ≤ 0.24%) (Fig. 1). Here, special care was taken that one whole fibre bundle was within the tapering of the specimen's shape (Fig. 1, red arrow B). Standard clamps were used for fixation. Initially, to ensure the same testing conditions at the beginning of each test, the specimens were preconditioned for 10 cycles ranging over 0–3% strain at a constant velocity of 10 mm/min. Then the tensile test was conducted at a strain rate of 2%/s until failure. During testing, the time, load and displacement were recorded and the *F*_*max*_, displacement at maximum force *s*_*max*_ in mm and the linear elastic modulus *E* were assessed. Here, the linear elastic modulus was defined as the slope in the linear region of the stress-strain diagram using linear regression.

**Indentation test:** To compare the biomechanical properties of the silk fibroin scaffold with literature data, an experimental test setup was used as previously published by Sandmann et al. (2013), the first and only research group, who evaluated the pre-implantation biomechanical properties of the two clinically available meniscal replacement devices CMI^®^ (Ivy Sports Medicine) and Actifit^®^ (Orteq Ltd.).

Cylindrical samples (n = 9) were cut from silk fibroin flat sheets indicated above (initial height h_0_: 5.12 ± 0.26 mm) using an 8-mm biopsy punch. The specimens were mounted in the materials testing machine, centred on a steel plate within a custom-made testing chamber filled with PBS. Furthermore, the setup for the indentation test included a calibrated 50-N load cell (KAP-S, A.S.T. GmbH, Blaustein, Germany, accuracy ≤ 0.28%) and a laser distance sensor (optoNCDT 2200-20, Micro-Epsilon, Ortenburg, Germany) to determine scaffold deformation. After applying an initial preload of 0.5 N, the cylindrical silk fibroin specimens were exposed to 5 load cycles using a steel-ball indenter (Ø = 5 mm). One cycle comprised a loading phase up to 7 N at 5 mm/min with a subsequent relaxation time of 60 s, followed by a load-release to 0.1 N at a constant velocity of 1 mm/min and finally deformation was held constant for another 60 s (recovery phase).

**Fig. 2:**
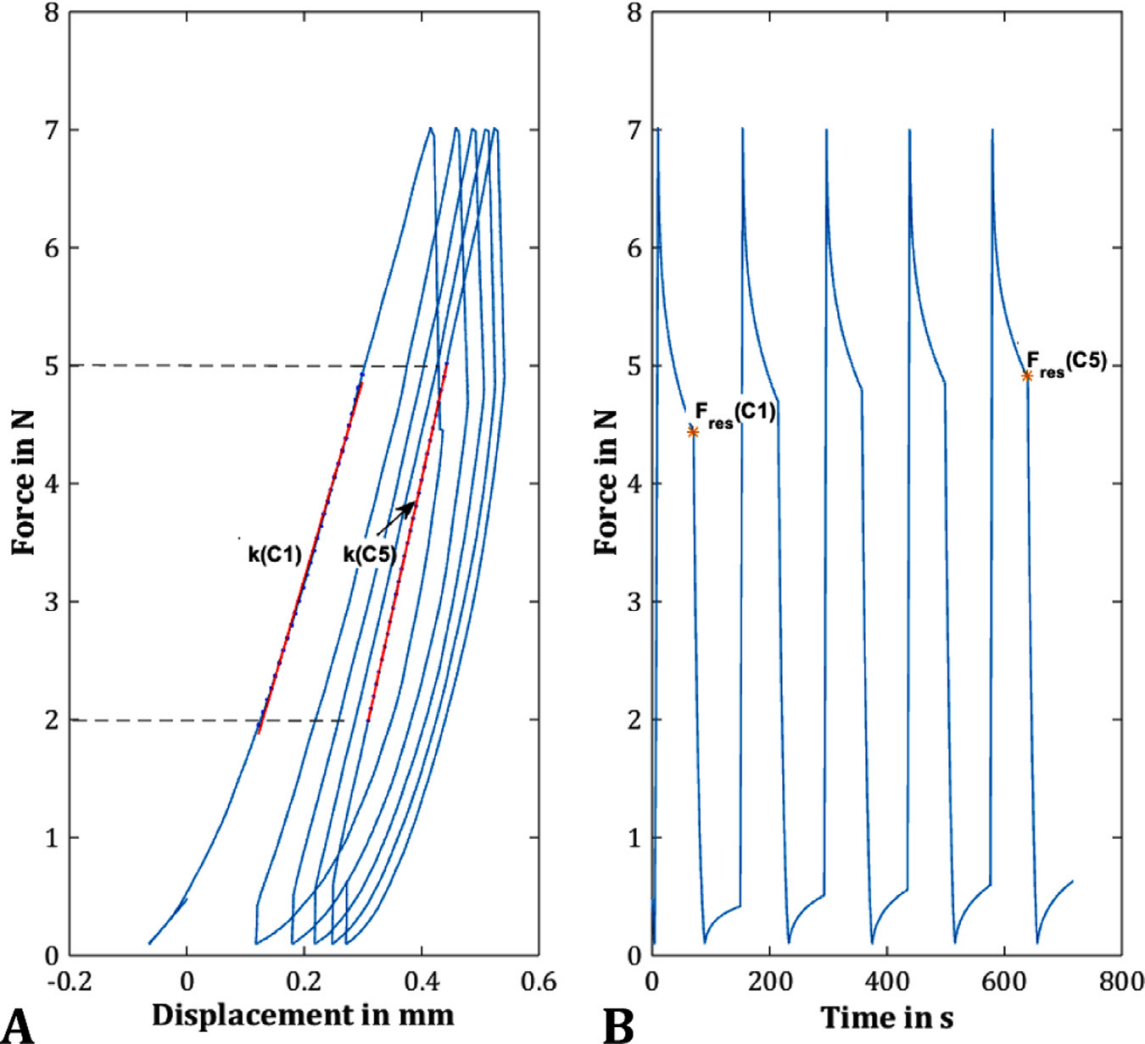
Representative curve of an indentation test, which comprised 5 load cycles up to 7 N with a subsequent relaxation time of 60 s, followed by a load-release to 0.1 N and another recovery phase. The stiffness in cycles 1 and 5 (k(C1) and k(C5), respectively) were determined from the linear region of the force-displacement diagram between 2 N and 5 N (A). As a measure for the viscoelastic behaviour, the residual force (F_res_(C1) and F_res_(C5)) were defined as the force remaining after the relaxation time at the end of the loading phase in cycle 1 and 5 (orange *, B). (For interpretation of the references to color in this figure legend, the reader is referred to the web version of this article.)

The stiffness *k* (N/mm) in cycles 1 and 5 was determined from the linear region of the force-displacement diagram between 2 N and 5 N (Fig. 2A). The *F*_*res*_, which was defined as the force remaining after the relaxation time at the end of the loading phase, was evaluated as a measure for the viscoelastic behaviour (Fig. 2B). Finally, the relative compression of the specimens in % was assessed by relating the recorded displacement of the indenter to the initial sample height.

**Unconfined compression relaxation test:** Unconfined compression tests were performed with a modified testing protocol according to Chia and Hull (2008), who determined the compressive properties of human menisci in axial and radial direction. Accordingly, cylindrical silk fibroin samples (n = 9) were obtained using a 5-mm biopsy punch. Within the materials testing machine, which was equipped with a 50-N load cell (KAP-S, A.S.T GmbH, accuracy: ≤ 0.28%), the cylindrical samples were mounted in a custom-made testing chamber filled with PBS and cyclically preconditioned to 12% strain for 10 cycles. Subsequently, a stress-relaxation test at 12% strain, controlled via a laser distance sensor (optoNCT 2200-2, Micro-Epsilon), was executed over a testing period of 60 min to ensure that an equilibrium state was reached. For evaluation, the *E*_*eq*_ was determined, which was defined by the quotient of the recorded stress at equilibrium *σ*_*t→∞*_ averaged over the last 10 min of testing time and the applied constant strain *ε*_*i*_ (1).

(1) $$E_{\mathrm{eq}}=\frac{\sigma_{t\to \infty }}{\varepsilon_{i}};\varepsilon_{i}=0.12$$

**Unconfined compression creep test:** To test the compressive behaviour of the silk fibroin scaffold under more physiological-like conditions, an unconfined compression test under creep conditions was performed. Here, the test setup was in accordance to Joshi et al. (1995), who quantified the differences in compressive properties of menisci of various species using the linear biphasic theory. Cylindrical samples of 5 mm in diameter, placed within a special testing chamber filled with PBS, were first preloaded to 0.02 N at a velocity of 1.6 mm/min for 15 min in the materials testing machine equipped with a 20-N load cell (Xforce P, Zwick GmbH & Co. KG, accuracy: ≤ 0.26%) and a laser distance sensor (optoNCDT 2200-20, Micro-Epsilon). Subsequently, the samples were loaded to 0.1 N at a velocity of 31% h_0_/min. This force was held constant for 60 min and the *E*_*eq*_ was determined (1).

##### 2.3.1.2 DMA

The dynamic loading measurements were performed using an ElectroForce^®^ 5500 dynamic materials testing machine (BOSE/TA ElectroForce Systems Group, New Castle, USA) equipped with a 200-N load cell (BOSE/TA ElectroForce Systems Group, accuracy: ≤ 1%) in an unconfined compression test setup. Additional cylindrical samples harvested using a 6-mm biopsy punch (n = 6; initial height h_0_ = 5.47 mm ± 0.12 mm) were mounted in the dynamic materials testing machine and preloaded to 0.2 N to ensure surface contact between the samples and the compression plates. Afterwards, a dynamic, sinusoidal strain with constant amplitude of approximately 60 µm was applied, passing through 5 cycles over a frequency spectrum of 0.1–10 Hz. The testing protocol used in the current study was based on Yan et al., who tested the material properties of silk fibroin scaffolds with different initial silk concentrations (Yan et al., 2012).

The standard software for the ElectroForce^®^ 5500 WinTest7^®^ continuously recorded the time, applied displacement and resulting force at a sampling rate of 100 Hz during testing. For a detailed characterisation of the silk fibroin scaffold, the loss factor tan *(δ)*, the storage modulus *E′* and the loss modulus E″ were evaluated from the first three recorded cycles of each frequency run.

### 2.4 Structural analysis: µ-CT

For structural analysis of the scaffold, µ-CT scans were executed with cylindrical samples punched out of 5-mm silk fibroin flat sheets, which had also been used for biomechanical testing. Because of the sample hydration, they first had to be dried. To preserve the geometrical dimensions and structure, a critical point drying method was chosen (E3100 Critical Point Dryer, LOT-QuantumDesign GmbH, Darmstadt, Germany). Prior to this, the samples were dehydrated through an ascending alcohol series (70%, 98% and 100%, each for 12 h).

Subsequently, the dried scaffolds were scanned (Skyscan^®^ 1172, Bruker microCT, Kontich, Belgium) with an approximate 8-µm image pixel size. The X-ray source was set at 40 kV and 250 µA. Projections were acquired over a rotation range of 180° with rotation steps of 0.36°. The reconstruction and analysis were performed using the standardised Skyscan^®^ software NRecon^®^ and CTAn^®^, respectively. All slices were converted to binary images with a threshold of 40–255 (grey values). To assess the microstructure of the silk fibroin scaffold, parameters like the total porosity, the mean pore size as well as the pore size distribution were evaluated.

### 2.5 Cell-culture experiments

#### 2.5.1 Scaffold preparation

Meniscus-shaped silk fibroin scaffolds were disinfected with 95% ethanol, washed three times with sterile PBS and pre-incubated for 24 h in standard culture medium (DMEM/Ham's F12, Gibco, ThermoFisher Scientific, Waltham, USA) containing 10% foetal calf serum (FCS) (PAA Laboratories, Cölbe, Germany), 1% penicillin/streptomycin (Gibco, ThermoFisher Scientific) 1% L-glutamine (Biochrom, Merck KGaA, Darmstadt, Germany), 10 µg/ml transferrin and 3 × 10^8^ M selenite (both Sigma-Aldrich, Taufkirchen, Germany).

#### 2.5.2 Cell cultivation

Chondrogenic murine ATDC5 cells, which were purchased from Sigma-Aldrich, and human mesenchymal stem cells (MSCs) isolated from bone-marrow aspirates as described previously (Mietsch et al., 2013) were used for the experiments. Briefly, the fresh human bone-marrow aspirates were obtained after informed consent and approval from the local ethical committee. Afterwards, the MSCs were isolated by density gradient centrifugation and plastic adherence. These types of cells were chosen since we assume that these cell types would have the closest contact to the scaffold material in vivo.

#### 2.5.3 Assessment of biocompatibility

Biocompatibility of the material was assessed by measuring cell metabolism and proliferation of both cells types separately cultured together with the scaffold material. Scaffolds were cut into square pieces (0.15 × 0.15 cm), which were placed in 96-well plates. A total of 200 µl culture medium containing 10,000 MSCs was added. The cells were cultivated for 1, 3, 14 or 21 days and cell metabolism was determined by the MTT test as described previously (n = 3 per time point) (Sarem et al., 2013). In a second experiment cell-proliferation was measured by a BrdU test according to the manufacturer's protocol (n = 6 per time point) using chondrogenic cells. For this, 200 µl culture medium containing 1000 ATDC5 cells was added to the scaffolds and cultivated for 7 or 14 days.

### 2.6 Statistics

Because the evaluated data were normally distributed (normal probability plot, Shapiro-Wilk test (Shapiro and Wilk, 1965)), the data were averaged and presented as means ± standard deviation. All further statistical analyses were performed using GraphPad Prism^®^ software (GraphPad Software Inc., La Jolla, USA).

1) The effect of cyclic indentation on the silk fibroin scaffold stiffness, residual force and compression were evaluated using paired Student's *t*-test.
  To compare these results with the existing data of Actifit^®^, CMI^®^ and human menisci obtained by Sandmann et al., additional one-way analysis of variances (ANOVA) with Bonferroni's multiple comparison tests were performed.
2) To determine any changes in the elastic/storage modulus *E′*, in the loss factor tan*(δ)* and in the loss modulus *E*″ depending on the frequency, one-way ANOVA with Tukey's multiple comparison tests were performed.
3) Within the biocompatibility test, changes in the cell metabolism and proliferation rate were analysed via one-way ANOVA and Student's *t*-test, respectively.

The statistical significance level was set to p < 0.05.

Due to the lack of comparability of the obtained data and the material analysis character of the study, all further obtained results were analysed descriptively.

## 3 Results and discussion

### 3.1 Biomechanical tests

#### 3.1.1 Tensile test to failure

**Fig. 3:**
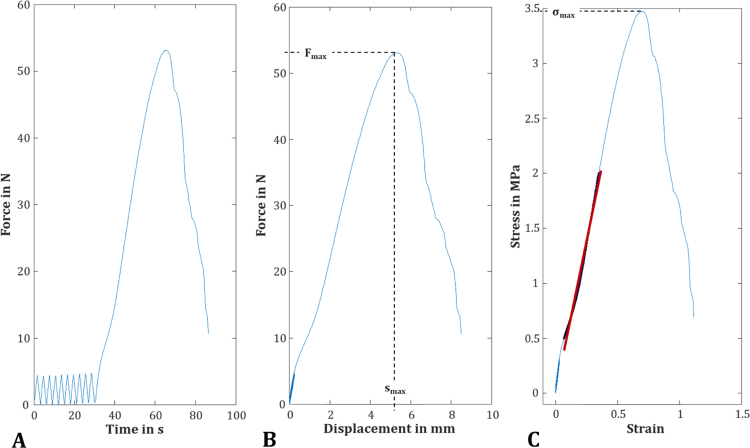
After 10-cycle preconditioning (A), the dumbbell-shaped samples of the silk fibroin scaffold were tested until failure at constant strain rate of 2%/s to determine the parameters F_max_, s_max_ (B) the ultimate tensile strength σ_max_ and the linear elastic modulus E out of the linear region of the stress-strain diagram (C; here: a representative curve).

Within this study, the tensile properties of a potential material for partial meniscal replacement were investigated for the first time using dumbbell-shaped samples (Fig. 3). They reached an ultimate tensile force of 51.0 ± 16.0 N at a maximum displacement of 4.7 ± 0.9 mm. Based on a rectangular cross-sectional area (length × width: 3.0 ± 0.1 mm × 5.1 ± 0.3 mm), the ultimate tensile force led to a maximum tensile strength of 3.28 ± 1.01 MPa. The mean elastic modulus, which was defined as the slope of the linear region of the stress-strain diagram, was 5.4 ± 1.4 MPa.

In general, there is a lack of information regarding the biomechanical characterisation of potential materials for (partial) meniscal replacement in the literature. Consequently, a comparison of the tensile properties of the silk fibroin scaffold with other materials was not possible. Therefore, data for the tensile properties of human menisci were consulted. Since the material properties of the meniscus are anisotropic, there are differences in the elastic modulus depending on the orientation of the collagen fibres (Tissakht and Ahmed, 1995, Lechner et al., 2000). Tissakht and Ahmed (1995), who determined the tensile characteristics of human menisci in two directions, found an almost 10-fold higher elastic modulus in the circumferential than in the radial direction (radial: lateral meniscus 11.6 MPa, medial meniscus 9.9 MPa; circumferential: lateral meniscus 111.7 MPa, medial meniscus 83 MPa). Comparing the properties of the scaffold with the literature, it is clear that the elastic modulus of the silk fibroin test samples was lower than that of native meniscus. However, the silk fibroin scaffold tested in the current study is designed to address partial meniscal replacement, in which the outer region of the meniscus and, therefore, the native circumferential collagen fibres are still maintained. Therefore, the functionality of transferring axial load into circumferential tensile stress is still provided in this scenario (Masouros et al., 2010, Beaupre et al., 1986). Furthermore, within the inner two-thirds, the menisci are predominantly exposed to compressive loads (Beaupre et al., 1986, McDermott et al., 2010), which leads to the assumption that the compressive properties of a potential material for partial meniscal replacement might be more important than its tensile properties. The integrated single layer of fibre mesh, which was implemented to enhance the fixation to the remaining host meniscus rather than to take up circumferential loads was arranged in an orthogonal array in the scaffolds. Therefore, it is possible that the scaffold may be improved by adopting a higher density of fibres with orientation that better mimics the circumferential arrangement of the collagen fibres found in the native meniscus especially for larger partial or for total meniscal replacements.

#### 3.1.2 Indentation test

**Fig. 4:**
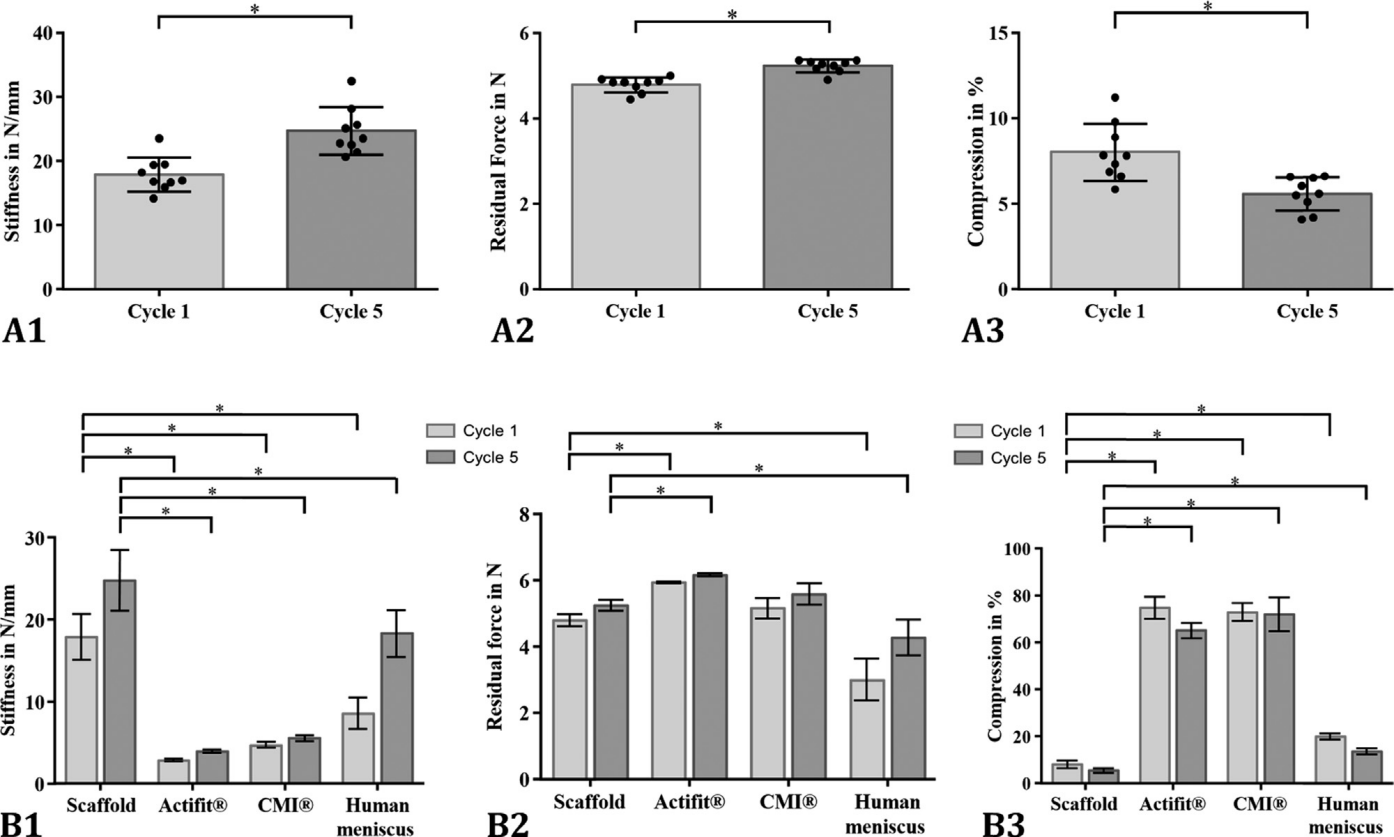
Mean and standard deviation of the silk fibroin scaffold's stiffness k in N/mm, residual force in N and their increase throughout the testing duration and the resultant compression rate at a 7 N load in the indentation test (cycle 1 vs. cycle 5, A 1–3, *p < 0.05). For a better comparison of these three parameters with the existing data of Sandmann et al. (2013), additional statistical analysis was performed (B1–3, *p < 0.05).

The indentation stiffness of the silk fibroin scaffold increased significantly between cycles 1–5 by approximately 38% from 17.9 ± 2.7 N/mm to 24.7 ± 3.7 N/mm (Fig. 4A1; p < 0.0001, two-tailed p-value). The residual force as a parameter for the viscosity also significantly increased from 4.8 ± 0.2 N in cycle 1–5.2 ± 0.1 N in cycle 5 (Fig. 4A2; p < 0.0001, two-tailed p-value). Consequently, the compression significantly decreased during test cycles 1–5 from 8.0 ± 1.7% to 5.6 ± 1.0% (Fig. 4A3; p = 0.002, two-tailed p-value within a paired *t*-test).

Attempting to find suitable test setups to compare the biomechanical properties of the silk fibroin scaffold with other meniscus replacement materials was difficult, because most investigators only reported histological analysis or gross examination via magnetic resonance imaging within in vivo studies (Stone et al., 1997, Maher et al., 2010, Verdonk et al., 2011). Therefore, we chose the test setup for a cyclic indentation test performed by Sandmann et al. (2013), the only research group investigating the biomechanical properties of the two clinically available implants CMI^®^ and Actifit^®^ in comparison with meniscus tissue of different species. They found significant differences in the viscoelastic properties and stiffness of both artificial materials in comparison to the native meniscal tissue (Fig. 4B1 and B2). It is clear that the silk fibroin scaffold was significantly stiffer in the first and fifth cycles not only compared to the two other artificial materials but also to human meniscal tissue (Fig. 4B1) but displayed fifth cycle average values that more closely approached meniscal tissue than either CMI^®^ and Actifit^®^. Similar differences were found in the compressive strain of the materials at a 7 N load (Fig. 4B3) with the silk fibroin scaffold, however, demonstrating average values that were closer to those of human meniscal tissue than the other two scaffolds. The F_res_ of the scaffold, defined by Sandmann et al. as a measure for the viscoelastic behaviour, was statistically significantly different in comparison to Actifit^®^ but also to human meniscal tissue also for both cycles 1 and 5 (Fig. 4B2). Consequently, the silk fibroin scaffold displayed initial compressive competence as required by Rongen et al., 2014, Stone et al., 1997 unlike the CMI^®^ and Actifit^®^. Sandmann et al. (2013) speculated that the low stiffness of these two implants might increase after implantation because of matrix deposition by ingrowing cells within the artificial materials, which was confirmed by Tienen et al. (2006) for a prior material version of the Actifit^®^ implant. Here, the increased stiffness was detected within an unconfined compression test after 3 and 6 months of implantation compared to the preoperative conditions. Nevertheless, the mechanical properties were still significantly different from native meniscal tissue. After 24 months, no further improvement of the mechanical properties occurred (Tienen et al., 2006). Rather, some scaffolds were totally destroyed, leading to considerable cartilage degeneration, contradicting the speculation by Sandman et al. (Welsing et al., 2008, Hannink et al., 2011).

#### 3.1.3 Unconfined compression relaxation and creep tests

**Fig. 5:**
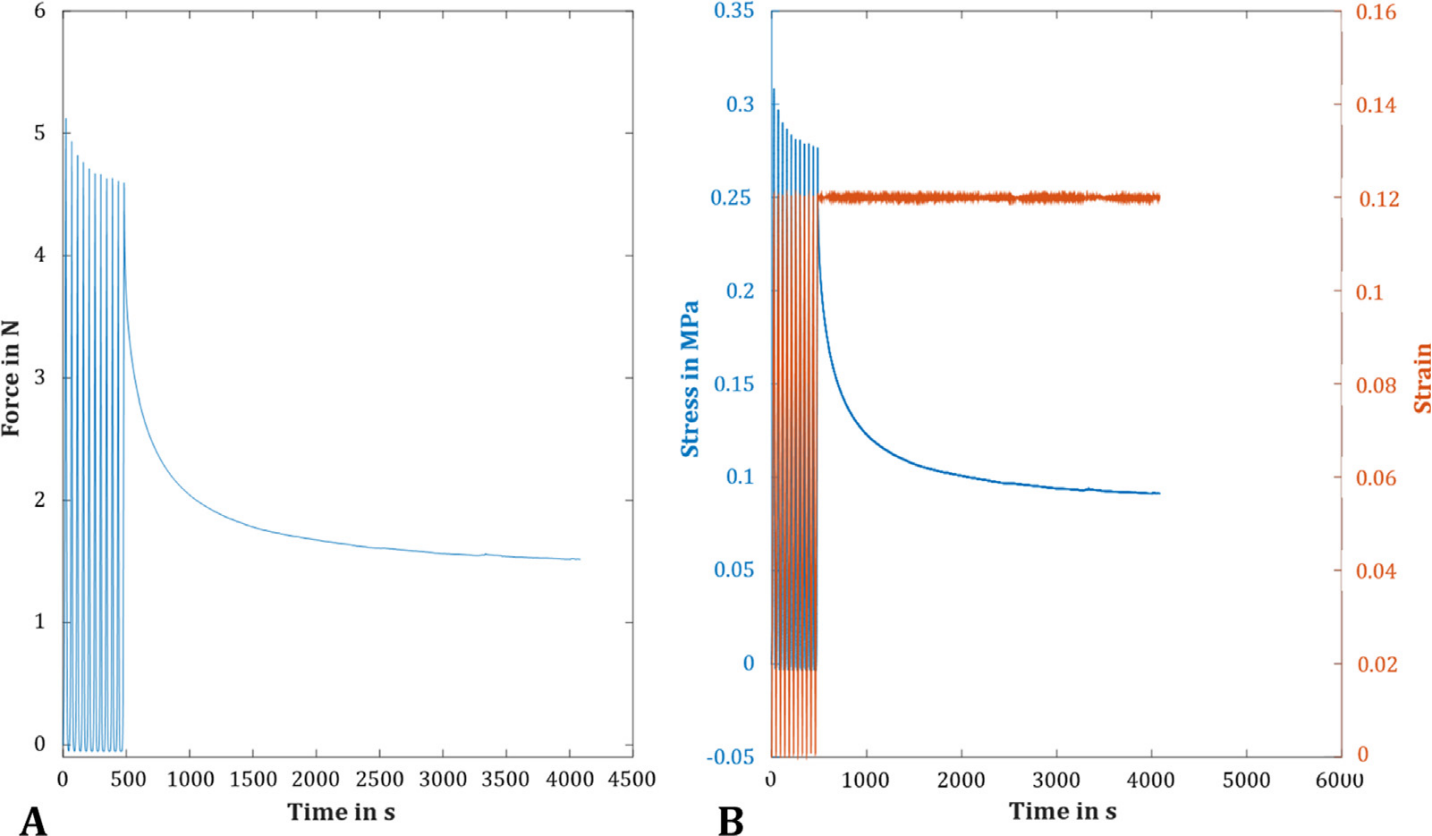
A representative curve of the decreasing force when performing an unconfined compression relaxation test at a physiological strain level of 12%, which was held constant for 1 h (A). The resultant stress (B, blue) was averaged over the last 10 min and divided by the applied strain (red) to determine the equilibrium modulus E_eq_. (For interpretation of the references to color in this figure legend, the reader is referred to the web version of this article.)

Based on the test setup of Chia and Hull (2008), we performed an unconfined compression relaxation test at a physiological strain level of 12% (Fig. 5). The resultant equilibrium modulus, representing the elastic properties of a viscoelastic material, was 560 ± 310 kPa.

This was stiffer than the compressive modulus at equilibrium of human medial menisci determined by Chia and Hull via a nonlinear least-squares regression using Fung's two-parameter exponential model. They assessed the highest equilibrium modulus to be approximately 138 kPa in the anterior region of the meniscus in the axial direction. However, such low values might partially be attributable to the extremely small sample dimensions of the tested 2 mm cubes. Samples of this dimension possibly do not reflect the properties of the largely inhomogeneous meniscal tissue correctly.

Gruchenberg et al., 2015, Gruchenberg et al., 2018 investigated a previous version of the silk fibroin scaffold in a sheep model, evaluating the equilibrium modulus preoperatively and 3 and 6 months post implantation and compared it with ovine meniscal tissue. They performed inter alia a stress-relaxation test at 20% strain and found a significantly higher equilibrium modulus for meniscal tissue (approximately 750 kPa) than for the scaffold (approximately 420 kPa) (Gruchenberg et al., 2015, Gruchenberg et al., 2018). In addition to the lower stiffness, the results of Gruchenberg et al., 2015, Gruchenberg et al., 2018 showed an insufficient fixation of the scaffold with the remaining meniscal tissue. Therefore, a new fibre layer was integrated within the porous matrix to enhance the surgical fixation. However, the authors postulate that the increased stiffness of the silk fibroin scaffold tested in the current study compared to the previous version may be due to this new fibre layer.

**Fig. 6:**
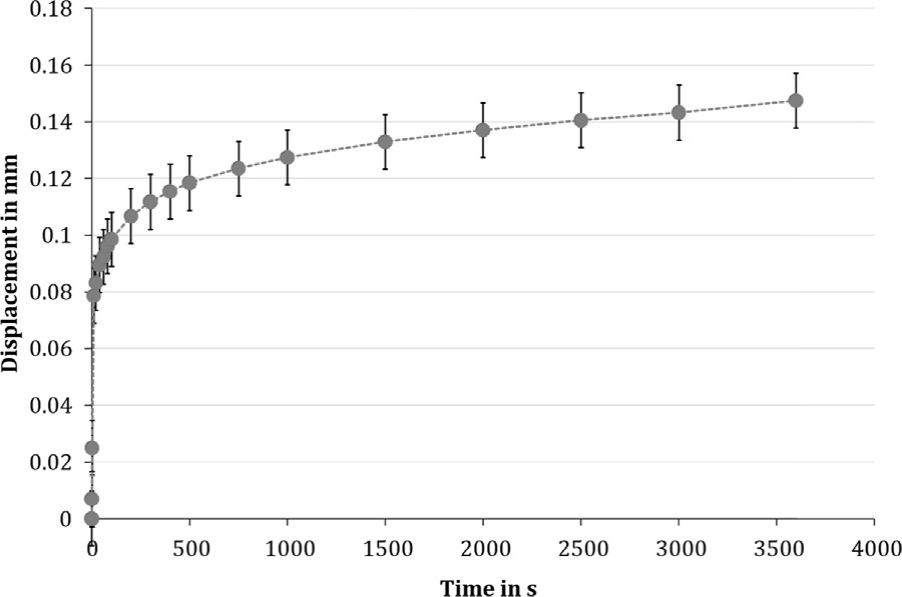
The unconfined compression creep tests of the silk fibroin scaffold revealed a typical viscoelastic creep response of the material with a mean deformation of approximately 2.2% (here: the averaged creep curve for all tested samples, n = 9).

To observe the material's behaviour under more physiological conditions, unconfined compression creep tests were also performed. Here, we adapted our test setup to that of Joshi et al. (1995), who quantified the compressive biomechanical properties of menisci of various species. The silk fibroin scaffold displayed a typical viscoelastic creep response, with a mean deformation of approximately 2.2% (0.15 ± 0.05 mm, Fig. 6) after 1 h. The equilibrium modulus was 0.30 ± 0.12 MPa. Joshi et al. determined an aggregate modulus H_A_ in MPa, which reflects the stiffness of the extracellular matrix, using the linear biphasic model. They found an aggregate modulus of approximately 0.2 MPa for human menisci (Joshi et al., 1995). The highest modulus was found for porcine meniscal tissue (approximately 0.27 MPa), which was, however, not significantly different to human menisci but even less than that of the silk fibroin scaffold.

Merriam et al., 2015, Patel et al., 2016 investigated a fibre-reinforced scaffold with two different material compositions (p(DTD DD) vs. PLLA, respectively) for total meniscal replacement in a sheep model. For mechanical characterisation of the scaffolds, they performed inter alia an unconfined compression creep test preoperatively and 16 and 32 weeks after implantation. They also used Mow's biphasic theory to evaluate the aggregate modulus of their collagen scaffold (p(DTD DD) fibres) and its modification (PLLA fibres), respectively (Merriam et al., 2015, Patel et al., 2016). They found that their scaffolds preoperatively reached an aggregate modulus of only 25% of the native meniscus (approximately 0.2 MPa versus 0.8 MPa for native ovine meniscal tissue), which, however, doubled after 16 weeks of implantation time. Despite the still existing discrepancy of the values between the p(DTD DD) fibre implant and the ovine meniscal tissue, the authors looked forward to a successful implant for total meniscal replacement due to the greater protection of the articular cartilage compared to a meniscectomy (Merriam et al., 2015).

The testing protocol according to Joshi et al. provided small loads of only 0.1 N, which may represent a limitation also of the current study. However, these low load magnitudes are necessary to ensure the assumption of the infinitesimal linearity of the linear biphasic model used by Joshi et al. (1995) as well as by Merriam et al., 2015, Patel et al., 2016. Nevertheless, to the best of the authors’ knowledge, the indicated studies are the only ones testing (different) menisci and also a potential meniscal replacement material under compression creep conditions.

#### 3.1.4 Dynamic mechanical analysis

**Fig. 7:**
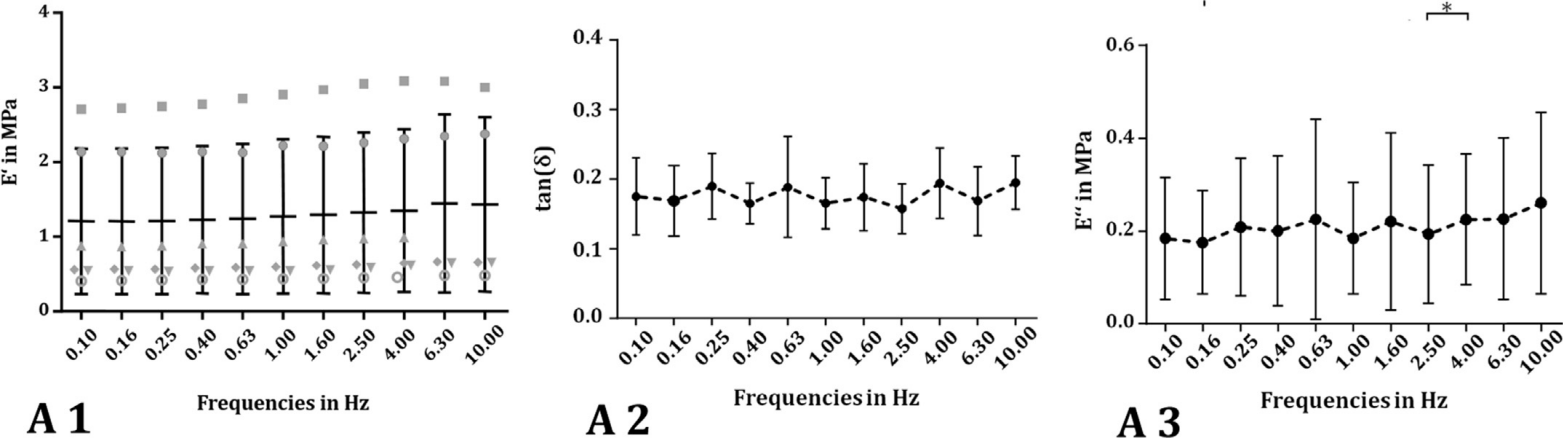
Within the dynamic mechanical analysis (DMA), a sinusoidal stress σ (or strain ε) is applied over a frequency spectrum and the material's response strain ε (or stress σ) is recorded. Because of the viscoelastic properties of the material, the response lags behind the applied stress (strain) with a phase-lag angle δ. For detailed characterisation of the silk fibroin scaffold, the dynamic elastic or storage modulus E' in MPa (A 1), the loss factor tan(δ) (A 2) and the so-called loss modulus E″ in MPa, as a parameter for the amount of energy dissipated by the viscous mechanisms (A 3), were evaluated.

With increasing frequency, the storage modulus *E′* of the silk fibroin scaffold did not increase significantly. Only a slight tendency of a continuous increase in *E′* from 1.20 ± 0.97 MPa at 0.1 Hz to 1.43 ± 1.17 MPa at 10 Hz could be identified (Fig. 7A1). This leads to the assumption that the energy, which is stored within the scaffold due to the elastic part of the material did not increase significantly with increasing loading frequency. The damping factor tan* (δ)*, which was calculated from the phase lag angle *δ* between the applied strain and the resultant material stress response, did not depend on the testing frequency (0.1 Hz*:* tan*(δ)* = 0.18 ± 0.06 and 10 Hz: tan* (δ)*= 0.19 ± 0.04; one-way ANOVA; Fig. 7A2), as well. Consequently, the damping properties are uninfluenced by the testing frequency, as well.

Comparing the obtained data with Yan et al. (2012), who investigated the dynamic compressive properties of silk fibroin scaffolds prepared with four different initial silk concentrations, with the scaffold tested in the current study, we found the material tested in this study to be two- to three-fold stiffer. However, the damping properties were quite similar to Yan's scaffold, particularly at a silk concentration of 12 or 16 wt%, whereas the dissolved fibroin concentration of the scaffold tested in the current study was 20%. Pereira et al. (2014) also determined the dynamic compressive properties of fresh human menisci, performing a DMA. They similarly found a tendency for an increased E’ with increasing frequency for medial and lateral menisci. However, the E’ was slightly different for both sides and also varied between the anterior, mid body and posterior regions. The mid body of the medial meniscus was stiffest and displayed elastic moduli between 0.83 MPa and 0.93 MPa for 0.1 Hz and 10 Hz, respectively, which was in the range of the silk fibroin scaffold tested in the current study (Pereira et al., 2014).

Additionally, we evaluated the loss modulus *E*″ as a parameter for the energy, which is dissipated by the viscous mechanisms within the material. Here, it was clear that *E*″ was also uninfluenced by the varied frequencies, comparable to the loss factor tan* (δ)*, whereby *E*″ ranged from 0.18 ± 0.13 MPa to 0.26 ± 0.19 MPa at 0.1–10 Hz, respectively (Fig. 7A3).

Comparing these data with human meniscal tissue, the silk fibroin scaffold tested in the current study had a slightly higher capability to dissipate energy than in the studies of Bursac et al., 2009, Pereira et al., 2014, as indicated by the higher loss modulus. Therefore, the elastic properties of the silk fibroin scaffold were more pronounced than its viscous character. Consequently, the amount of energy dissipated by the viscous mechanisms was minor compared to the energy stored within the material because of the elastic components.

### 3.2 Structural analysis: µ-CT

**Fig. 8:**
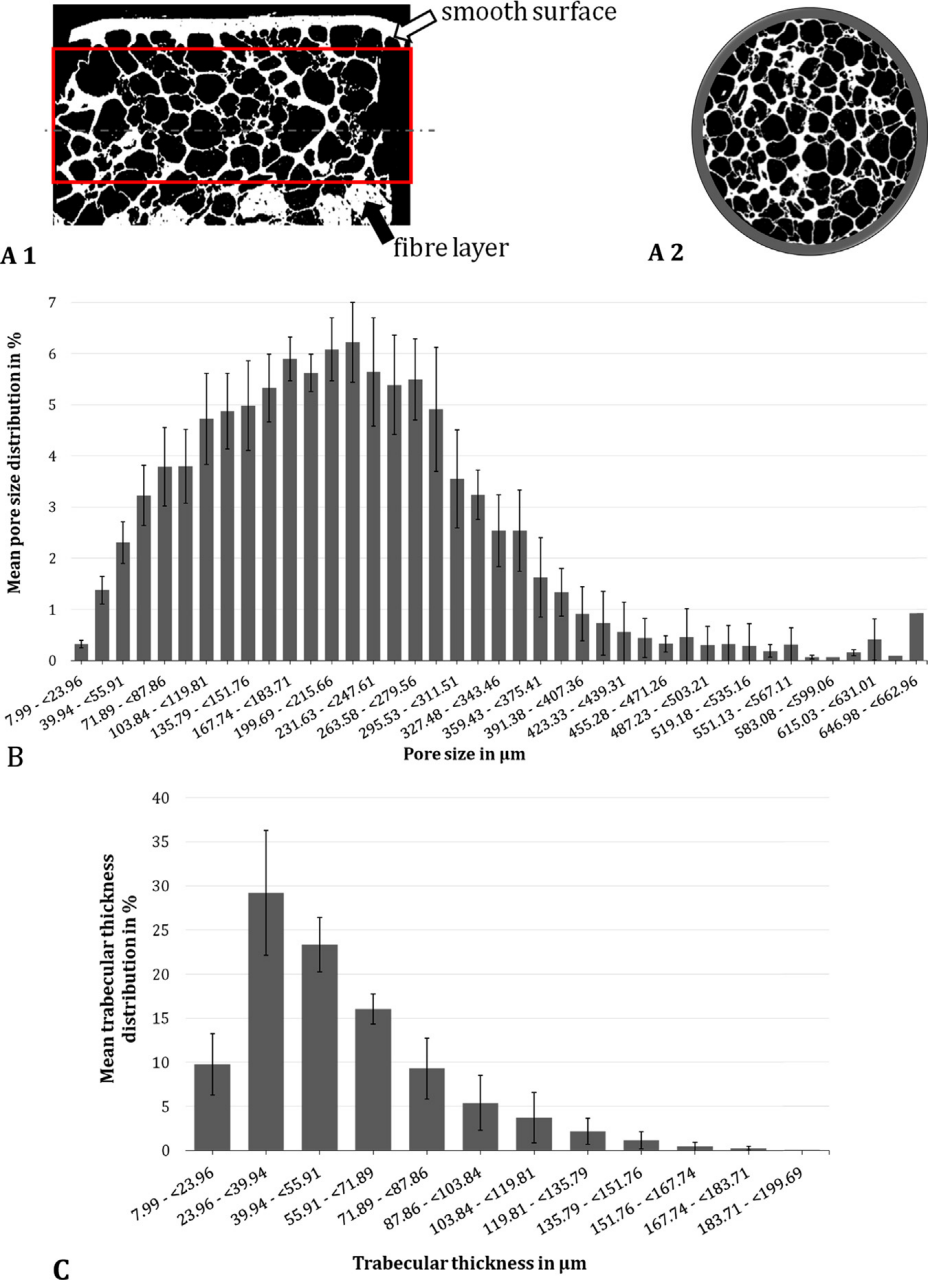
Exemplary sagittal (A 1) and transversal (A 2) µ-CT images of a critical point dried silk fibroin scaffold. From µ-CT analysis, the mean and standard deviation of the pore size distribution (B) and the distribution of the trabecular thickness (C) were evaluated (n = 5).

The structural composition and architecture of the silk fibroin scaffold was determined by µ-CT analysis (Fig. 8). Here, a mean volume of 24.2 ± 6.87 mm³ of each sample was analysed. The variations in the analysed volume arose from the location of the integrated fibre layer (Fig. 8), which was excluded from the analysis to prevent any misinterpretations of the ultrastructure of the scaffold. µ-CT analysis revealed a total porosity of 80.13 ± 4.32% (open porosity: of 80.12 ± 4.32%), with a mean pore size of 215.6 ± 10.9 µm. In general, the pore size distribution was Gaussian, but slightly shifted to the left. However, it ranged over 8–663 µm, with more than 65% of the pores being 100–300 µm in diameter (Fig. 8). A mean pore wall thickness of 53.6 ± 9.6 µm was observed, with almost 30% of the pore walls measuring 24–40 µm (Fig. 8).

Microstructural parameters, including pore size and total porosity, are crucial for replacement materials, because they can affect not only the integration but also the regeneration of new meniscal tissue. Therefore, Rongen et al. included these parameters in their requirements for meniscal replacement materials. They suggest having both large macropores (200–300 µm) in turn connected by smaller micropores (10–50 µm), resulting in a high interconnectivity. Additionally, one should aim for a high total porosity of > 70% (Rongen et al., 2014). Comparing the obtained values in the present study with these requirements, it is clear that the silk fibroin scaffold fits well with these guidelines for structural composition of meniscal replacement materials. ﻿

### 3.3 *In vitro* biocompatibility test

**Fig. 9:**
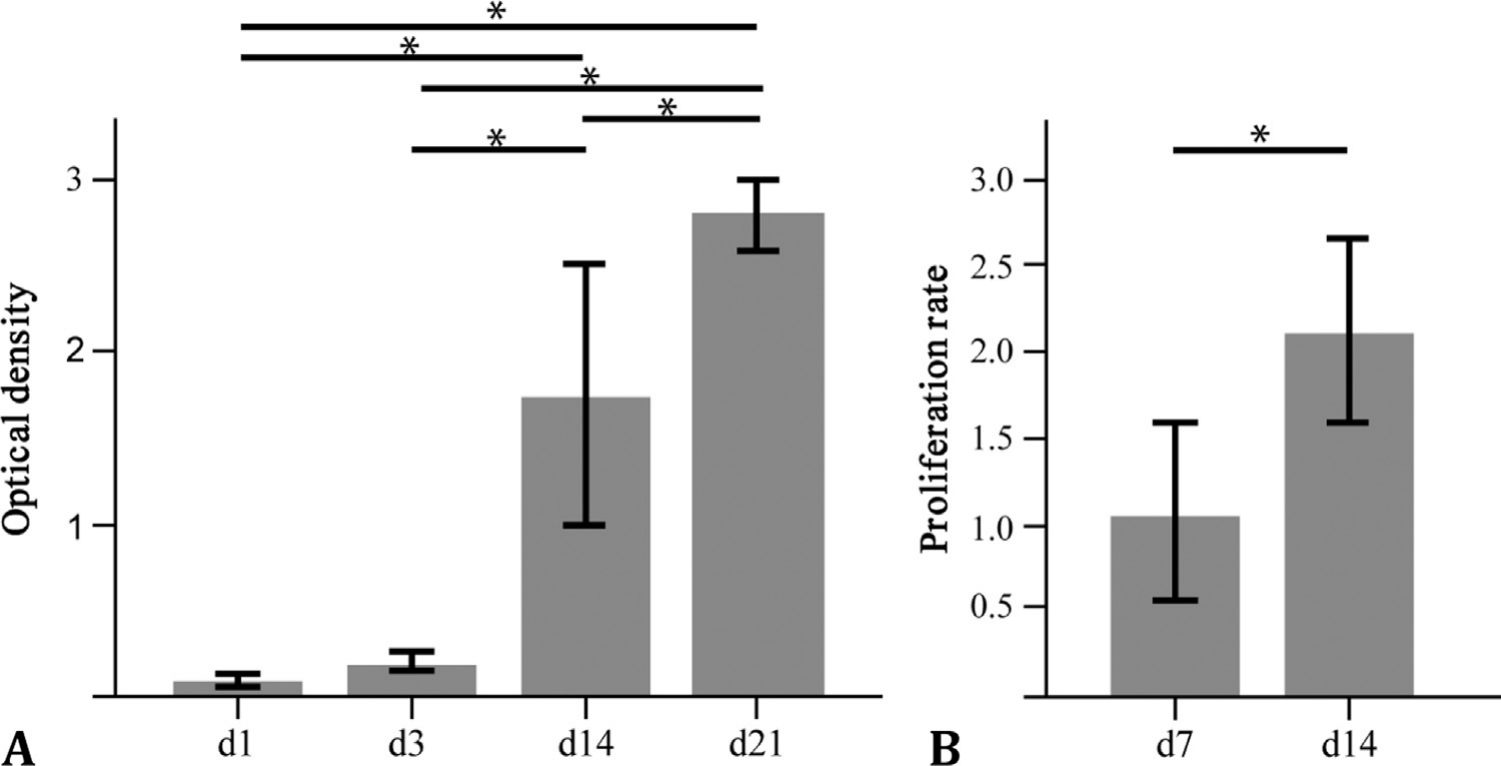
Assessment of the biocompatibility (A, B) of the silk scaffold. Therefore, ATDC5 cells and human MSCs were co-cultured with the silk fibroin scaffold and the cell metabolism of MSCs was assessed by the MTT test (C), whereas the cell-proliferation rate of ATDC5 cells was assessed by the BrdU test (D). (n = 3–6) *p < 0.05.

The MTT and BrdU tests demonstrated undisturbed cell viability and increasing proliferation over time in the presence of the scaffold material, indicating a sufficient biocompatibility of the material, with no toxic side effects on co-cultured cells (Fig. 9A and B). Therefore, we conclude that the scaffold material does not have a general negative effect on cell proliferation and metabolic activity.

Because of its unique material properties, silk has been used decades long in biomedical applications. Combined high mechanical strength and favourable elasticity make silk a suitable suture material (Altman et al., 2003). Silk extracted from the silkworm cocoon (*Bombyx mori*) mainly consists of two fibroin proteins, which are encased with a sericin coat. Thereby, sericin serves as a “glue” to hold the two core fibres together (Altman et al., 2003). The immunogenicity of silk-based biomaterials arises from the sericin coating of the fibroin proteins, because isolated fibroin proteins did not activate the immune system (Panilaitis et al., 2003). Therefore, the biocompatibility of isolated fibroin has been demonstrated in various in vitro and in vivo studies (Santin et al., 1999, Meinel et al., 2005, Cassinelli et al., 2006, Seo et al., 2009). During the manufacturing process of the silk fibroin scaffold used in the current study, fibroin was extracted and processed into a porous matrix. Biocompatibility of the first generation of this scaffolds was previously confirmed in an in vivo study using a partial meniscal replacement model (Gruchenberg et al., 2015). Furthermore, there was insufficient fixation stability resulted in less integration of the scaffold into the remaining meniscal tissue, leading to displacement of the material during the experimental period in a third of all cases. Therefore, this study revealed the need to improve scaffold fixation to the meniscal rim (Gruchenberg et al., 2015) and a fibre mesh was accordingly inserted into the porous matrix. The current study showed that the material properties in terms of biocompatibility did not change during this process, therefore the second generation of this material can be used in an in vivo setting in the future.

## 4 Conclusion

Within the current study, we characterised the mechanical and structural properties as well as the biocompatibility of a second generation, new, silk fibroin scaffold as a potential material for permanent partial meniscal replacement (Table 1).

**Table 1:** Summary of all biomechanical measurements and evaluated data achieved during tensile-, indentation-, unconfined compression relaxation and creep tests and during dynamic mechanical analysis (DMA).

**Tensile test**	**Ultimate tensile force F**_**max**_	51.0 ± 16.1 N
	**Ultimate tensile strength**	3.28 ± 1.01 MPa
	**Elastic Modulus E**	5.37 ± 1.45 MPa
	**Displacement at ultimate force s**_**max**_	4.75 ± 0.92 mm
**Indentation test**	**Stiffness k**	
	Cycle 1	17.9 ± 2.69 N/mm
	Cycle 5	24.7 ± 3.73 N/mm
	**Residual force F**_**res**_	
	Cycle 1	4.79 ± 0.17 N
	Cycle 5	5.23 ± 0.15 N
	**Compression**	
	Cycle 1	8.03 ± 1.68%
	Cycle 5	5.58 ± 0.97%
**Unconfined compression**	**Relaxation (Equilibrium modulus Eeq)**	0.56 ± 0.31 MPa
	**Creep (Equilibrium modulus Eeq)**	0.30 ± 0.12 MPa
**DMA**	**Frequency range**	0.1–10 Hz
	**Storage/elastic modulus E′**	1.20 ± 0.97 MPa–1.43 ± 1.17 MPa
	**Loss factor tan(δ)**	0.18 ± 0.06–0.19 ± 0.04
	**Loss modulus E″**	0.18 ± 0.13 MPa–0.26 ± 0.19 MPa

Many approaches have been published to restore rather than resect the injured meniscus (Rongen et al., 2014, Rodkey et al., 1999, Buma et al., 2004, Kohn et al., 1992, Walsh et al., 1999, Bruns et al., 1998, Gastel et al., 2001, Peters and Wirth, 2003, Verdonk et al., 2005, Milachowski et al., 1990, Noyes and Barber-Westin, 2005). Only two artificial substitutes (CMI^®^ and Actifit^®^) are used clinically, but these are still not widely accepted by medical professionals. Additionally, their pre-operatively mechanical properties do not approach those of human meniscal tissue as Sandmann et al. showed significant differences in the viscoelastic properties of both artificial replacement concepts in comparison to human menisci. Long-term biomechanical data of these implants are not available in the literature.

Rongen et al. (2014) further elaborated the basic requirements for meniscal replacement materials first postulated by Stone et al. (1997). It is important for a replacement material to support mechanical loads already in the initial phase following implantation and that it should mimic the native meniscus as closely as possible (Stone et al., 1997, Rongen et al., 2014). Considering these requirements, the silk fibroin scaffold for partial meniscal replacement tested in the current study displayed a sufficient compressive competence although slightly in excess of the native human meniscus. The material showed an increased stiffness in comparison to the first scaffold generation, which is likely to be associated with a fibre component integrated in the scaffold to enhance fixation strength to the meniscal host tissue. These new fibres did not provide comparable tensile strength to native meniscus but, because the inner region of the meniscus, which is the target for a partial meniscal replacement, is more exposed to compressive rather than to high tensile loads (Masouros et al., 2010, Beaupre et al., 1986, McDermott et al., 2010). Nevertheless, it is expected that the scaffold could be improved, especially for larger replacement defects, by adopting a higher density of fibres with orientation, which better mimics the circumferential orientation of the collagen fibres of native meniscal tissue. Although the silk fibroin scaffold presented mechanical competence, which is important to distribute loads over the articulating surfaces and therefore, reducing peak stresses already in the initial phase after implantation, a too stiff construct may impede tissue ingrowth and additionally damage the underlying articular cartilage (Rongen et al., 2014). Maher et al. (2010) also indicate that the transition zone between implant and meniscal host tissue is always a critical region as the mismatch in stiffness could inhibit the success of partial meniscal replacement materials. Therefore, adjusting the structural parameters, like pore size distribution and pore wall thickness, may present an opportunity to reduce the material's stiffness to more closely simulate the biomechanical/compressive properties of menisci, even if they were in the range of the requirements postulated by Rongen et al. (2014). Initial mechanical competence is important and desirable for load transmission and therefore, chondroprotection even in the early postoperative phase. However, addressing this mechanical capability over the full range of meniscal tissue loading is important in developing a stable implant-tissue interface. Given the characteristic anisotropy and inhomogeneity of meniscal tissue, this will always be a major challenge to replicate. Nevertheless, we conclude that varying the ratio and orientation of the stiffer fibres in the silk fibroin scaffolds tested in the current study to a more compressible porous hydrogel matrix may offer a route to better emulating the full range of native meniscus properties.
